# Multidimensional vulnerability and financial risk protection in health in contexts of protracted conflict: Evidence from the Occupied Palestinian Territory

**DOI:** 10.1371/journal.pone.0314852

**Published:** 2025-01-16

**Authors:** Julia Hatamyar, Sally Shayeb, Akseer Hussain, Weeam Hammoudeh, Sumit Mazumdar, Rodrigo Moreno-Serra

**Affiliations:** 1 Centre for Health Economics, University of York, Yorkshire, United Kingdom; 2 Institute of Community and Public Health, Birzeit University, West Bank, Palestine; 3 Faculty of Public Health, Al-Quds University, West Bank, Palestine; Aix-Marseille Universite, FRANCE

## Abstract

This paper proposes a multidimensional vulnerability index for a setting of protracted conflict, which is applied to study the relationship between financial vulnerability and catastrophic healthcare expenditure (CHE) incidence in the Occupied Palestinian Territory in 2018. We find that our index better captures the extent of financial risk protection in health compared to conventional measures of financial welfare. Results indicate that the most vulnerable groups experience a significantly higher likelihood of incurring CHE, and this likelihood is increased for those living in the West Bank compared to the Gaza Strip. We also find a lack of protection from existing health insurance types against the risk of CHE. Our analysis provides valuable insights about key aspects, such as health financing and insurance bottlenecks, that will deserve careful policy attention in efforts to rebuild the Palestinian health system, following the Israel-Hamas war.

## Introduction

In settings with protracted conflict and other humanitarian challenges, conceptualisation of household vulnerability and its association with financial risk protection (FRP) in health may require a broader perspective than in more stable and peaceful areas. Conventional welfare measures such as household income or consumption expenditure, generally used in health financing research, may not fully capture the multi-faceted sources and aspects of vulnerability present in these unique settings, where threat of violence, political turmoil, forced displacement and movement restrictions can play a large role in individual well-being and financial risks. In such settings, measures of economic capacities need to be supplemented with appropriate, objective indicators from other contextually-relevant dimensions of vulnerability. Indicators that capture insecurities caused by the conflict in key areas of human security and welfare—food, water, shelter, freedom of movement, risks of injury, serious trauma and disabilities—are crucial to conceptualisations of vulnerability and realistic assessments of FRP. Furthermore, conventional indicators of FRP based on income- or expenditure-centric measures of welfare, such as catastrophic spending incidence, also have well-known limitations for adequately capturing the welfare impacts of excessively high health payments in low-income and low health coverage settings more generally [1]. Consequently, as we aim to illustrate, conventional FRP measures may significantly underestimate the risks and mask the welfare effects of household vulnerability to high spending on health care, particularly in humanitarian settings.

In this paper, we develop a multidimensional index of household vulnerability for populations experiencing protracted conflict, which we argue is more appropriate to measure the risk and welfare consequences of inadequate FRP in health in such settings, than conventional indicators. Such multidimensional indices can be used to inform equitable health policies, identify specific subgroups experiencing difficulties, and respond to health emergencies [2, 3]. We apply our index in the Occupied Palestinian Territory (OPT) and examine its relationship with catastrophic health expenditure. The OPT exhibits a complicated political and economic situation [4]. Across the West Bank, Gaza strip and East Jerusalem, the occupied Palestinian territory (OPT) has witnessed protracted conflict and associated humanitarian consequences since 1948 [5]. Population health has faced detrimental consequences arising from extended periods of colonisation, along with other challenges, notably the fragmentation of the Palestinian health system and a reliance on international aid [6–8].

Using our multidimensional index, we observe how financial risk protection varies between the two major regions of the OPT—the West Bank (WB) and the Gaza Strip (GS)—and across other stratifying characteristics (e.g. sub-regions within West Bank, type of locality, education/occupation). We also aim to contribute to literature seeking to adjust FRP measurement in contexts with low access to healthcare services. In areas such as Gaza, which are more heavily affected by conflict violence and have even poorer health care infrastructure, we observe how such restricted access affects the demand for health care, consequently depressing health care expenditure. We identify several questions for future research, paving the way for deeper investigation into the complexities of healthcare access and FRP in challenging contexts.

Throughout this paper, we conduct analysis for the OPT as a whole, and also separately for WB and Gaza. As the two regions exhibit significantly different characteristics (see Methods Section), we wish to examine whether the relationship of vulnerability with CHE also differs across regions. However, we also wish to capture broader aspects of financial risk protection in areas of protracted conflict, which in this setting comprises the shared “Palestinian experience”; therefore some composite measure of FRP which is consistent across the two regions (our vulnerability index), is a desirable property.

Our study also aims to contribute to the literature seeking to adjust FRP measurement in contexts with low access to healthcare services. In areas such as Gaza, which are more heavily affected by conflict violence and have even poorer health care infrastructure, we observe how such restricted access affects the demand for health care, consequently depressing health care expenditure. Our broader multidimensional measurement approach is amenable to adaptations in the specific sources of household vulnerability considered, which makes it generally useful for providing a more rounded assessment of FRP in other settings of low access to health services, beyond Palestine and humanitarian contexts.

We begin with a brief overview of the Palestinian context and a review of the literature on financial vulnerability in conflict affected areas. In the following sections, we show that our composite vulnerability measure is capable of effectively capturing information about the likelihood of households incurring CHE, and explore the heterogeneous effects of vulnerability across regions, insurance type and status, and locality (urban versus rural dwelling, refugee camps).

### Background and motivation

#### Conflict affected areas and health services

Conflict, whether internal or international, is a crucial social determinant of health outcomes [9], directly and indirectly jeopardising access to essential services [10–12], appropriate nutrition, and healthcare facilities [13–16]. Conflict often does not end with clear outcomes like victory or peace agreement; instead, it frequently leads to a state of limbo with fighting simply ceasing [17]. The transition to a post-conflict situation is hardly ever linear, and some nations can relapse into conflict [18, 19]. The protracted conflict has immediate consequences, but also long-term impacts influencing multiple generations. It can affect economic access, social stability, healthcare availability, and access to health facilities [20–22].

Conflict-affected states typically experience worse health service provision [18, 23, 24] compared to non-conflict-affected states [25]. This situation is exacerbated by factors like high out-of-pocket payments, low government health expenditure, and external dependency on funding [26]. Conflict-affected states encounter various challenges in delivering healthcare, including ideological and operational conflicts among service providers [27, 28]. They also face issues related to care for internally displaced persons and refugees, as the displaced suffer poor living conditions impacting health, a high burden of existing disease and injury from conflict, and limited access to primary care services [29]. Improving healthcare provision in these regions can be achieved through public financing, efficient utilisation of resources by governments [26], performance-based financing [30], subsidies [31], and community involvement [32]. These measures are essential to advance universal health coverage and address health disparities prevalent in these challenging environments.

#### Overview of the palestinian conflict and health system

The formation and structure of the Palestinian health system is deeply rooted in its complex political history. The OPT have been in political turmoil and under protracted conflict for many decades, with implications for public services such as health, education and social protection [7]. Since the Oslo Agreement of 1993, which aimed to achieve peace between the Israeli government and the Palestine Liberation Organisation (PLO) and led to the establishment of the Palestinian Authority (PA), there has been noticeable decline in Palestinian economic standards, worsening social degradation, and an acceleration of the de-development process (The concept of “de-development” in the Palestinian context refers to a process through which infrastructure and economic capacity have regressed or stifled, rather than simply underdeveloped) [5, 33]. These issues are compounded by unenforced international human rights legislation, internal Palestinian divides, and regular confrontations between Israeli and Palestinian armed organisations [34]. One of the outcomes of the protracted conflict between the governments of Israel and Palestine is enclavisation, i.e. the physical separation of the West Bank and the Gaza strip [5, 35]. The resulting absence of a long-term, unified political settlement and prospects for future development weakened an already frail public-sector structure, particularly in health-care delivery [34].

From 1967, following the occupation of the OPT and until 1995, the Israeli Civil Administration managed the governmental health care system, which was consistently underfunded and limited in terms of health and social services coverage [7, 36]. Severe budget constraints, referral to Israeli hospitals for tertiary care, and restrictions on licenses for new healthcare projects resulted in the OPT’s total reliance on the Israeli health system [4]. Following the Oslo Accords, health governance responsibilities were partially transferred to the newly established Palestinian Authority (PA) in 1994, which attempted to build a more comprehensively structured health system and established the Ministry of Health [37]. Between 1994 and the onset of the Second Intifada in 2000, efforts were made to standardise health services, improve health infrastructure, and train healthcare professionals. International donor assistance increased during this brief period, allowing for further development of healthcare infrastructure, though new challenges were introduced by inconsistency in funding and complex coordination efforts required to distribute aid. The renewal of conflict during the Second Intifada (2000–2005) severely impacted the nascent healthcare system, causing damage to the new infrastructure, restricting movement and thereby access to healthcare, increasing the medical needs of the population [38]. Local systems were unable to handle these needs, increasing reliance on referrals to outside healthcare providers [39].

Since the seizure of the Gaza strip by Hamas in 2007, the West Bank and Gaza have been under separate political leaderships, the PA and Hamas, and Israel began blockading the Gaza strip with land, sea, and air closures. This has severely limited the movement of people and goods in and out of the area—including healthcare professionals, patients in need of complex procedures unavailable within Gaza, and health supplies [7, 40, 41]. The geopolitical split has led to a fragmented health system between the two regions, with challenges in governance, resource allocation, and health strategy coordination. Decreased tax revenue and foreign aid created financial instability, worsening the ability of the PA to maintain healthcare services [8, 42]. These conditions comprised the baseline of health and social services prior to the Israel-Gaza war beginning in October 2023.

Despite PA efforts toward establishing adequate social protection [43, 44], there is an anticipated rise in the rate of poverty across the life cycle in the OPT [43]. Governmental and non-governmental assistance vary in coverage and impact. Additionally, vulnerable groups such as individuals with disabilities are not adequately addressed by existing social protection policies [43]. Furthermore, health inequalities are the result of a complex interplay of variables including economic difficulties, food shortages, environmental exposures, psychological trauma and stress, and limited access to health care. Most of these factors may be directly or indirectly linked to Israel’s military occupation of the OPT [45]. In 2018, the year of focus in this study, there were 299 Palestinians killed and 31,723 injured in occupation-related violence, of which the majority occurred in the Gaza strip. In addition, violent attacks on healthcare workers, equipment and facilities impacted public access to healthcare services, with 432 such attacks occurring in 2018 (WHO Report, “Right to Health in the occupied Palestinian territory: 2018”). The frequency of attacks on medical missions is correlated with peaks in occupation-related injuries and fatalities [46].

### Conceptualising vulnerability in protracted conflict settings: Health financing, FRP, and CHE

The assessment of socioeconomic inequities encountered by disadvantaged communities is especially important in protracted conflict settings. Previous work has shown that creation of multidimensional indices for use in the study of health expenditure and other outcomes can provide benefits beyond use of individual measures alone in making these assessments. Pinilla-Roncancio et al. [47] create a multidimensional poverty index using a modified Alkire-Foster technique [48], capturing information such as access to clean water, sanitation facilities and electricity, as well as aspects related to mitigation of negative health shocks, such as asset ownership and employment status. The authors then examine its relationship with CHE in seven different LMICs—importantly, each country requires its own unique index depending on heterogeneous characteristics and data availability—and show that CHE incidence can drive long term poverty. In Palestine, such an index has been used by the Bureau of Statistics and included both monetary and non-monetary elements, to expand the scope of poverty studied, but without connecting the index to health expenditures (although information such as disability prevalence, presence of chronic diseases, health insurance and health facility accessibility are used in creation of the index) [49].

Without basic human security, international aid directed towards provision of healthcare in Palestine is unable to promote its development [40]. Vulnerability to shocks of a more fundamental and physical nature than financial poverty is more likely to impact risk protection in health in this context. Direct threats to security faced by Palestinians, such as restrictions on movement, arrests and detentions, gunfire, home demolition, injury and death, will not be captured in a poverty index. Survey data (see Methods Section) show the collective history of mass displacement and being repeatedly uprooted have resulted in commonly experienced feelings of insecurity and instability. Therefore, in this paper we develop a composite vulnerability index that aims to capture complex dynamics of CHE, which is often related to poverty but may encompass other aspects in settings of protracted conflict. Examining CHE through this index can assist in measuring household financial resilience and coping methods in the face of healthcare payments, better reflecting the degree of FRP—or lack thereof—among the most vulnerable groups in a long-term conflict scenario.

## Methods

### Data and variables

In this study the empirical analyses are based on data from the 2018 Socio-Economic Conditions Survey conducted by the Palestinian Central Bureau of Statistics (PCBS) in coordination with the Food Security Sector (co-led by the FAO and WFP), supported by the United Nations Relief and Works Agency (UNRWA) and the Union of Agriculture Working Committee (UAWC). SEFSec-2018 is the third round of the panel survey, which includes respondents from 9926 families.

The outcome variables of interest in our analysis are catastrophic health expenditures (CHE) as a percentage of household consumption expenditure (at the 10% threshold), and household non-food expenditure (at the 20% threshold). The 10% gross expenditure threshold is commonly used by global organisations to assess FRP and monitor progress towards UHC [50], and is also used in earlier work examining CHE incidence in the OPT [51]. Table S1 Table depicts overall CHE incidence across a range of thresholds varying from 5–40%, and shows that for the non-food expenditure version, the differences across the two regions are stronger and more significantly different. This motivates our use of the 10% consumption expenditure outcome in both the main and secondary analyses. The main variable of interest that we use to examine variation in the CHE outcome is the *vulnerability index*.

To create the vulnerability index, a factor analysis was performed using a number of survey questions capturing both objective and subjective measures (described below). Although the use of factor analysis does not allow for direct interpretation of the individual determinants of CHE [52], our aim in this paper is to reduce dimensionality in the data and create a single index as an exploratory mechanism, in line with previous work (for example, [53]). We do not use any rotation when performing the analysis, as primary goal is not to interpret the factors. The factor analysis is done separately for each region, West Bank and Gaza (as factor loadings may be different, given the significant regional differences shown in Table 1), and a composite vulnerability score is predicted from these factors. Using the principal factor method in Stata, factors are retained if their eigenvalues of the covariance matrix exceed the value of 1. In this step only a single factor met the eigenvalue threshold, so we also performed a parallel analysis which confirmed the choice to retain only the first factor [54, 55]. Supplemental Materials S1 Fig depicts a scree plot of the eigenvalues and the results of the parallel analysis. Our vulnerability index was finally obtained by assigning scores to each observation. These scores are weighted averages of the covariates, with weights determined by the factor loadings (coefficients indicating the strength of the association between each variable and the factor).

**Table 1:** Summary statistics by region.

**Socioeconomic Variables**	WB N = 5895	Gaza N = 4025	Mean Diff (ttest)	Prob > |*z*| (Wilcoxon)
Not Working	0.264	0.496	-0.23***	0.000
Part-Time Work	0.103	0.128	-0.02***	0.000
Full-Time Work	0.338	0.211	0.13***	0.000
Long Work Hours	0.296	0.165	0.13***	0.000
Elementary or Less Education	0.319	0.253	0.07***	0.000
Preparatory Education	0.326	0.279	0.05***	0.000
Secondary Education	0.160	0.183	-0.02**	0.004
Above Secondary Education	0.195	0.286	-0.09***	0.000
No Disability/Chronic Condition	0.715	0.638	0.08***	0.000
Chronic Condition Only	0.143	0.119	0.02***	0.000
Disability Only	0.061	0.141	-0.08***	0.001
Chronic and Disability	0.081	0.102	-0.02***	0.000
No Insurance	0.299	0.064	0.23***	0.000
PA only	0.379	0.270	0.11***	0.000
UNRWA only	0.126	0.162	-0.04***	0.000
PA+UNRWA	0.116	0.493	-0.38***	0.000
Urban	0.692	0.847	-0.16***	0.000
Rural	0.243	0.000	0.24***	0.000
Camps	0.065	0.153	-0.09***	0.000
HH members	4.737	5.621	-0.88***	0.000
Dependency Ratio	0.740	0.881	-0.14***	0.000
Healthcare Facility Access Difficulty	0.193	0.270	-0.08***	0.000
Received Any Assistance	0.091	0.676	-0.59***	0.000
Refugee	0.266	0.615	-0.35***	0.000
**Vulnerability Index Components**	WB	Gaza	Mean Diff	Prob > |*z*|
Self-Assessed Poverty	0.103	0.478	-0.37***	0.000
Self-Assessed Financial Fragility	0.207	0.643	-0.44***	0.000
Subjective Need for Asssistance	0.442	1.456	-1.01***	0.000
Political Conflict Shock	0.122	0.042	0.08***	0.000
Economic Shock	0.203	0.610	-0.41***	0.000
Health/Education Shock	0.131	0.541	-0.41***	0.000
Freedom Shock	0.027	0.102	-0.07***	0.000
Water Shock	0.323	0.537	-0.21***	0.000
Food Security Shock	0.196	0.797	-0.60***	0.000
Asset Ownership Index	2.364	1.600	0.76***	0.000
Subjective Deprivation	1.437	2.666	-1.23***	0.000
Human Insecurity Scale	36.006	41.422	-5.42***	0.000
**CHE Measures**	WB	Gaza	Mean Diff	Prob > |*z*|
10% Consumption Exp.	0.352	0.274	0.08***	0.000
20% Non-food Exp.	0.134	0.103	0.03***	0.000

This table presents the mean values of variables used in the analysis across WB and Gaza, as well as their difference in means.

* *p* < 0.10,

** *p* < 0.05,

*** *p* < 0.01 for a ttest of difference in means.

Prob >|*z*| is the value from a non-parametric Wilcoxon rank-sum test (Mann-Whitney) of difference in distributions.

Our vulnerability index is similar in spirit to the concept of human insecurity explored in Palestine by Ziadni et al. [41], who describe insecurity comprising two aspects: basic material needs required for survival, and a psychological/social component [56]. Factors used in our vulnerability index composition are: self-assessed economic status, self-assessed household resilience (whether a household is able to keep up financially), self-assessed need for public (financial) assistance, and a combined shocks experience variable which includes political, economic, health, educational, freedom, water availability, and food-related shocks, asset ownership (calculated using principal component analysis), self-assessed deprivation level, and a human insecurity scale. We report details of the survey questions used, including the scale of variables, in Supplemental Materials S1 Appendix. Table in S2 Table shows the vulnerability index eigenvalues, variance explained, and factor loadings for both regions, and indicates differences in loadings between the two regions. For example, both regions have similar loading magnitudes for poverty status, need for assistance, and subjective deprivation, but the loadings for financial fragility and human insecurity are much higher in Gaza compared to the West Bank. To ensure the correct choice of variables in the creation of the factor index, we performed a LASSO regression to check whether any of the covariates would not be chosen for inclusion (i.e., would have coefficients shrunk to zero), which confirmed that all of the chosen shocks should be included. LASSO results are reported in S12 Table For the main analyses, terciles of vulnerability composite index are used (we also use quintiles of the index to validate the results, see Supporting information). For multivariate analyses, we include relevant covariates, such as employment status categories, education level of household head, presence of disability or other chronic health conditions, health insurance status and type; and number of household members. Details regarding these covariates can be found in the S1 Appendix.

Table 1 shows mean-differences and p-values by region for all covariates. The Shapiro-Wilk and Skewness-Kurtosis tests of normality, as well as the Bartlett’s test for equal variances, indicate that a nonparametric test is most appropriate; we therefore report p-values from the Wilcoxon rank-sum test in the final column. The two regions of the OPT differ immensely in terms of healthcare, educational, and occupational characteristics, likely due to the closure of nearly 80% of industry in Gaza after the Israeli blockade [41]. Compared to West Bank residents, individuals living in Gaza are more likely to be unemployed, have more disabilities and chronic conditions, and live in urban areas and refugee camps. Gazans are much more likely to self-assess themselves as fragile, poor, and in need of financial assistance. They face larger economic and food security shocks by orders of magnitude compared to West Bank residents, who face larger political conflict shocks.

**Fig 1:**
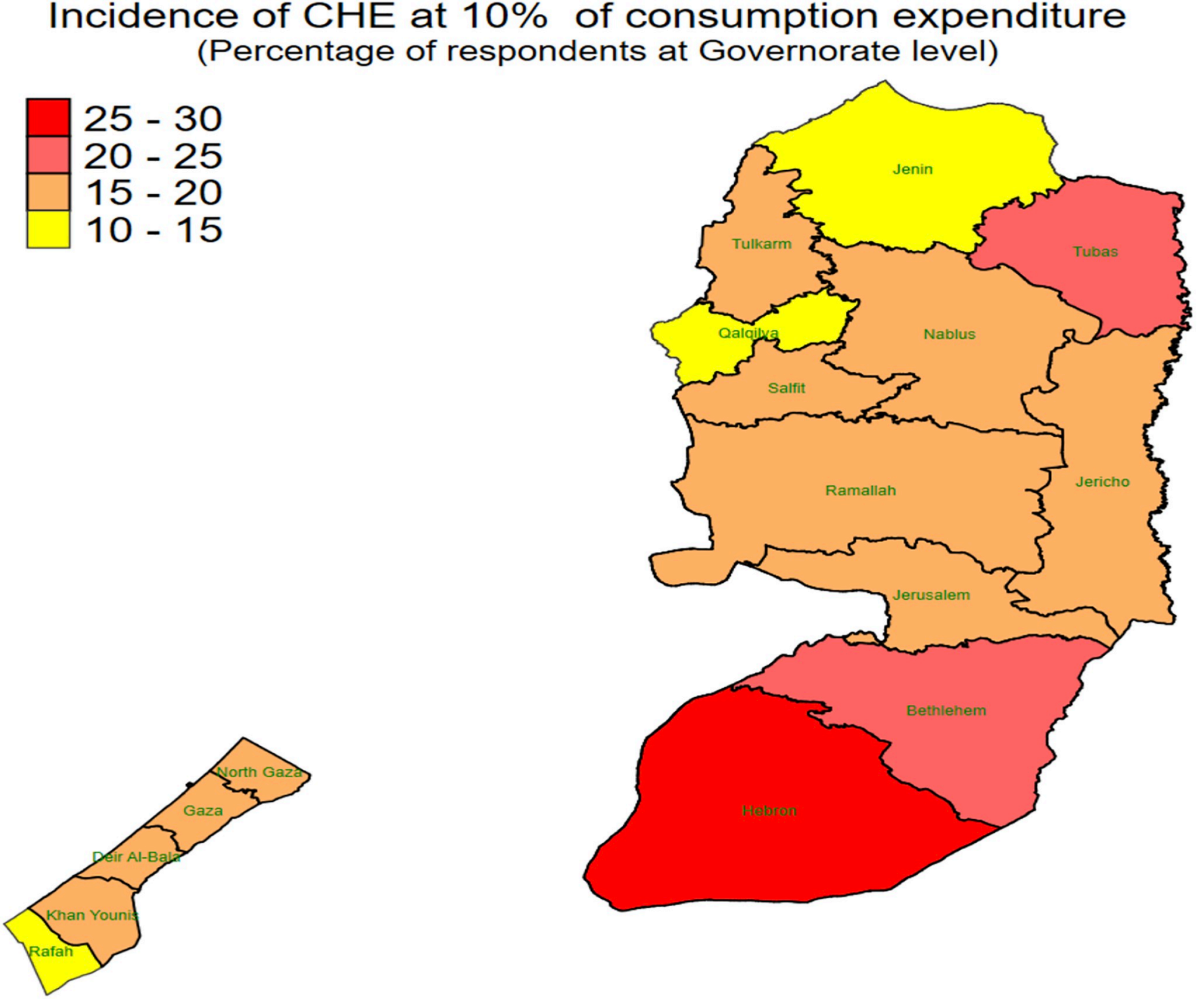
CHE by governorate. This figure depicts the incidence of CHE at the 10% level across the governorates of the OPT. The map was created by the authors using shape-files publicly available by United Nations Office for the Coordination of Humanitarian Affairs (https://data.humdata.org/dataset/cod-ab-pse?), using Stata software. The data used for CHE calculation was taken from https://mas.ps/en/sefsec.

We also present information on overall incidence of CHE at various thresholds ranging from 5% to 40% of consumption and non-food expenditure and of total household expenditure by region in S1 Table. Trends across the varying thresholds are similar to those in previous work on both the OPT and in developing countries [51, 57]. We note that, regardless of threshold or definition of living standard/capacity to pay, CHE incidence is higher in the West Bank than in Gaza. Fig 1 visualises the intensity of the incidence of CHE at governorate levels. The map was created by the authors using shape-files publicly available by United Nations Office for the Coordination of Humanitarian Affairs, using Stata software, and data used for CHE calculation was taken from the SEFsec. It demonstrates clear geographic variation in incidence, with southern areas of the West Bank experiencing more CHE than northern West Bank. For example, in Hebron, located in the Southern WB, more than 25% of respondents have faced CHE at the 10% threshold compared to the northern governorates of the West Bank.

**Fig 2:**
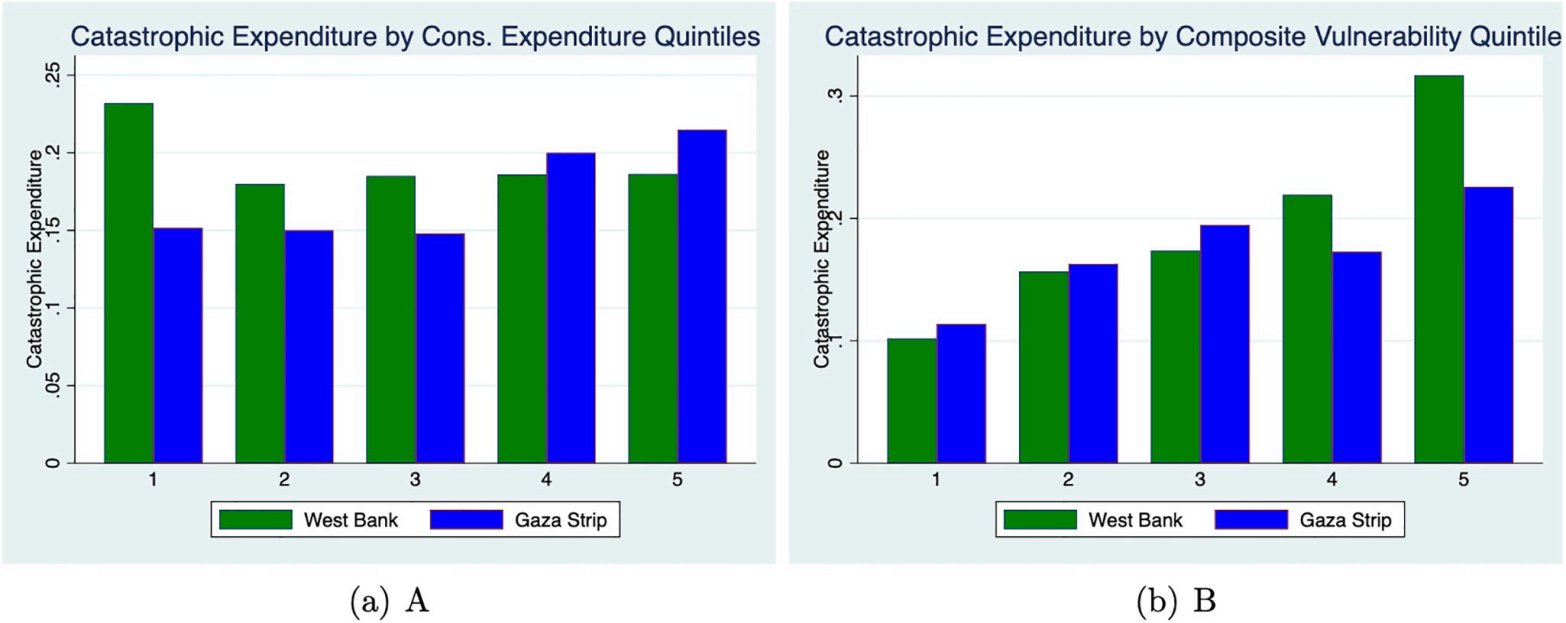
Catastrophic health expenditure comparisons. This figure compares incidence of CHE by consumption-expenditure quintiles (Panel A) and by the vulnerability index (Panel B).

Furthermore, we calculate Catastrophic Health Expenditure (CHE) at the 10% threshold by Consumption-Expenditure quintiles, and also by quintiles of our proposed vulnerability index (described in the Methods Section), using data from the 2018 Socio-Economic Conditions (SEFSec) Survey. Fig 2 visually demonstrates these CHE quintiles and shows that a conventional determinant of catastrophic health expenditure, overall consumption expenditure, may not accurately assess risk of CHE in the context of Palestine. There is a slight inverse relationship across the two regions of Palestine (WB and GS) between consumption expenditure and catastrophic health expenditure (Panel A). In WB, those with lower consumption expenditure (quintile 1) are most likely to experience CHE, while in Gaza, the highest two consumption expenditure quintiles are more likely to experience CHE. Conversely, our composite vulnerability measure is mostly rising with catastrophic expenditure in both regions (Panel B), and households in both WB and Gaza in the top vulnerability quintile are the most likely to experience CHE.

### Regression model

For analysing the relative risks of experiencing CHE across different levels of multidimensional vulnerability, we run the following logistic regression:

$$\begin{array}{c} y_{i}=\alpha +\beta_{1}1\left\{VulnerabilityTercile_{i}\right\}+\beta_{2}X_{i}+\nu_{i}+\epsilon_{i} \end{array}$$ (1)

where *y*_*i*_ is catastrophic health expenditure at the 10% threshold, **1**{*VulnerabilityTercile*} is an indicator for being in a tercile of the vulnerability index, *ν*_*i*_ is the governorate fixed effect, and *ϵ*_*i*_ is the error term [39, 58]. We choose a fixed effects model with governorate fixed effects to control for unobserved, time-invariant characteristics specific to each governorate, reducing potential bias from omitted variables that may differ across governorates. In the Supplemental Materials we report results from a variety of model specifications including random effects and generalised linear models. Results are robust to all of these alternatives and the information criteria (lowest AIC/BIC) indicate that the fixed effects model is indeed the best model choice in this setting. The choice of logistic regression assumes the error distribution is implicitly binomial with a logit link function. Although this analysis is seeking to uncover associations between the vulnerability index and CHE in the data, and not striving for strict causal identification, we still utilise a comprehensive group of control variables in the primary analysis, *X*_*i*_, including employment status, household size, insurance status and type, education level, and a variable indicating whether there is chronic illness or disability present in the household. This provides some reassurance that the associations estimated through our regression model are indeed indicative of *potential* causal links between the relevant variables and CHE. Detailed descriptions of covariates can be found in the Supporting Information.

## Results

Here we present the main results of this paper, which show the association between multidimensional vulnerability and the likelihood of incurring CHE. Our results are robust across specifications, with the most vulnerable groups being significantly more likely to experience catastrophic health expenditures. The likelihood is higher in WB compared to Gaza.

As indicated in Table 2 Panel A, Palestinian households within the second and third terciles of the vulnerability index are significantly more likely to incur CHE at 10% of total expenditure and at 20% of non-food expenditure. The most vulnerable households are 76.2% and 105.3% more likely to incur CHE at the mentioned thresholds, respectively. In both specifications, WB experiences a higher likelihood of experiencing CHE in the most vulnerable tercile. Panel B of Table 2 compares the impact of quintiles of monthly per-capita consumption expenditure (MPCE) with the impact of our vulnerability index on CHE at the 10% level of total expenditure. It is clear that the MPCE measure does not have a significant impact, nor any discernable pattern. For example, there is a slight impact of the fourth MPCE quintile in GS (OR 1.51), but the magnitude decreases for the highest quintile and does not remain significant. In contrast, our vulnerability largely shows a consistent increasing relationship with CHE. For the OPT as a whole and for the West Bank, the magnitude of coefficients is increasing with quintiles, and for GS the highest quintile also has the largest effect. We report goodness-of-fit measures and find that the pseudo *R*^2^ measure is consistent with, and in some specifications larger than, the pseudo *R*^2^ measure in comparable literature examining the determinants of CHE [59, 60]. We describe the effects of other (control) variables, which are often highly significant, in the Supporting Information.

**Table 2:** Catastrophic health expenditure and vulnerability.

**PANEL A: Consumption Expenditure vs. Non-food Expenditure**						
Outcome Variable:	CHE-10%			Non-food- 20%		
Odd Ratios:	(1)	(2)	(3)	(4)	(5)	(6)
	All—	WB	Gaza	All	WB	Gaza
**Index Tercile = 2**	1.264**	1.135	1.500**	1.455***	1.420**	1.552***
	(0.141)	(0.171)	(0.251)	(0.141)	(0.234)	(0.127)
**Index Tercile = 3**	1.762***	1.846***	1.617***	2.053***	2.085***	1.920***
	(0.144)	(0.216)	(0.148)	(0.241)	(0.255)	(0.447)
*Observations*	9647	5801	3846	9646	5800	3846
*Clusters*–*Governorate*	16	11	5	16	11	5
Log pseudolikelihood	-4155.259	-2486.087	-1654.208	-3149.32	-1939.402	-1196.529
Pseudo *R*^2^	0.097	0.120	0.066	0.121	0.152	0.070
**PANEL B: Consumption Expenditure vs. Vulnerability Index**						
Quintile Definition:	MPCE			Vulnerability Index		
Dep: Var: CHE-10%	(1)	(2)	(3)	(4)	(5)	(6)
	All	WB	Ghaza	All	WB	Gaza
**Index Quintile = 2**	0.917	0.834*	1.088	1.448***	1.447**	1.463*
	(0.089)	(0.086)	(0.173)	(0.181)	(0.272)	(0.289)
**Index Quintile = 3**	0.958	0.931	1.058	1.588***	1.484	1.782***
	(0.095)	(0.101)	(0.204)	(0.218)	(0.379)	(0.201)
**Index Quintile = 4**	1.068	0.893	1.515*	1.740***	1.851***	1.571***
	(0.143)	(0.118)	(0.338)	(0.126)	(0.214)	(0.125)
**Index Quintile = 5**	0.994	0.836	1.499	2.280***	2.486***	1.963***
	(0.160)	(0.115)	(0.462)	(0.232)	(0.398)	(0.161)
*Observations*	9887	5872	4015	9647	5801	3846
*Clusters*–*Governorate*	16	11	5	16	11	5
Log pseudolikelihood	-4270.14	-2531.531	-1721.291	-4148.84	-2482.197	-1652.808
Pseudo *R*^2^	0.091	0.115	0.062	0.098	0.122	0.067

Panel A compares two different dependent variables: CHE at 10% total expenditure and 20% of non-food expenditure. Panel B compares two independent variables: monthly consumption expenditure versus the vulnerability index. Exponentiated coefficients are shown with standard errors in parentheses, clustered at governorate level. Governorate fixed effects in all models. All control covariate coefficients and AIC/BIC measures reported in Supporting Information.

* *p* < 0.10,

** *p* < 0.05,

*** *p* < 0.01.

### Heterogeneity analysis

#### CHE by insurance type

Due to initial findings of a highly significant and large relationship between governmental health insurance and likelihood of incurring CHE (see regression coefficients in Supporting information), here we subset the data and repeat the analysis. First, we subset the data by insurance type, and then by insurance status. Results for regressions on data subsetted by insurance type and status are shown in Table 3. Coefficients for control variables are reported in the Supporting Information. For those individuals only with governmental (PA) insurance, we see that the impact of being in the highest vulnerability tercile is slightly larger than that of the entire sample (1.78 in Panel A, versus 1.76 in S4 Table), and the pattern is consistent across the two regions. Households with only governmental health insurance experience a statistically significant increase in the likelihood of CHE at 10% of total expenditure, signifying a 33.1% and 78.7% higher likelihood of incurring CHE in second and third vulnerability terciles (p<0.01). For Gaza, results show a 87.6% and 58.3% higher likelihood of incurring CHE among second and third vulnerability terciles, respectively. Also of note is the lack of any association of the vulnerability terciles with CHE for those individuals only possessing UNRWA insurance. This suggests that the impact of insurance type on CHE varies across vulnerability levels, with a stronger effect for those in the higher vulnerability category who are not in possession of UNRWA insurance.

**Table 3:** Catastrophic health expenditures, heterogeneity analysis.

**PANEL A: Insurance Type**						
Insurance Type:	PA Only			UNRWA Only		
Dep. Var: CHE-10%	(1)	(2)	(3)	(4)	(5)	(6)
	All	WB	Gaza	All	WB	Gaza
**Index Tercile = 2**	1.331**	1.133	1.876***	1.122	1.225	1.109
	(0.178)	(0.153)	(0.398)	(0.229)	(0.343)	(0.409)
**Index Tercile = 3**	1.787***	1.877***	1.583***	1.523	1.481	1.623
	(0.172)	(0.250)	(0.219)	(0.402)	(0.519)	(0.752)
*Observations*	3246	2212	1034	1352	728	624
Log pseudolikelihood	-1550.178	-1046.168	-496.7447	-513.2543	-269.3285	-240.6537
Pseudo *R*^2^	0.100	0.119	0.070	0.108	0.152	0.065
**PANEL B: Insurance Status**						
Insurance Status:	Insured			Uninsured		
Dep. Var: CHE-10%	(1)	(2)	(3)	(4)	(5)	(6)
	All	WB	Gaza	All	WB	Gaza
**Index Tercile = 2**	1.334**	1.121	1.620***	1.337*	1.337	1.312
	(0.172)	(0.168)	(0.300)	(0.226)	(0.236)	(1.010)
**Index Tercile = 3**	1.825***	1.820***	1.767***	2.622***	2.571***	2.339
	(0.172)	(0.307)	(0.157)	(0.356)	(0.343)	(2.540)
*Observations*	7670	4063	3607	1972	1736	236
Log pseudolikelihood	-3431.259	-1808.249	-1611.824	-769.8006	-706.5768	-56.89859
Pseudo *R*^2^	0.079	0.110	0.048	0.111	0.105	0.197
**PANEL C: Locality Type**						
Locality Type:	Urban			Rural		**Camps**
Dep. Var: CHE-10%	(1)	(2)	(3)	(4)		(5)
	All	WB	Gaza	All		All
**Index Tercile = 2**	1.403***	1.212	1.740***	0.987		1.115
	(0.157)	(0.161)	(0.271)	(0.245)		(0.179)
**Index Tercile = 3**	1.966***	1.979***	1.987***	2.077**		1.648**
	(0.112)	(0.184)	(0.136)	(0.591)		(0.384)
*Observations*	7253	4008	3245	1417		972
Log pseudolikelihood	-3179.821	-1767.409	-1398.255	-585.0798	-382.3032	
Pseudo *R*^2^	0.084	0.110	0.056	0.143	0.137	

Exponentiated coefficients; Standard errors in parentheses. SE clustered at governorate level, with governorate fixed effects in all models. All control covariate coefficients and AIC/BIC measures reported in Supporting Information.

* *p* < 0.10,

** *p* < 0.05,

*** *p* < 0.01.

#### CHE by insurance status

In Panel B of Table 3, we report for data subsetted by insurance status. We find that those who have any type of insurance are less likely than the uninsured to incur CHE. Further, likelihood of incurring CHE increases with vulnerability index terciles, by 33.4% and 82.5% for the second and third terciles among insured households and 33.7% and 162% among uninsured households. The most vulnerable groupsare more likely to incur CHE, regardless of health insurance status. Examining results by region, we find in WB, CHE risk increases by 82% among insured households (Panel B) compared to 87.7% among households with governmental health insurance only (Panel A). However, in the Gaza Strip, the most vulnerable tercile with *any* type of insurance is more likely to incur CHE than those with the PA type only (76% vs 58%).

In summary, the health insurance heterogeneity analysis suggests that association between health insurance type and the likelihood of incurring CHE is not uniform across vulnerability index terciles. The most vulnerable groups are more likely to incur CHE, regardless of their health insurance status. However, the type of health insurance may play a role in the likelihood of CHE for the most vulnerable groups.

#### CHE by locality type

We also compare results for data subsetted by locality type (urban, rural, and refugee camps). Results are shown in Table 3, Panel C. Results are consistent with the overall findings and show an increase in the likelihood of CHE as vulnerability increases, but also show that those living urban areas experience a greater likelihood of incurring CHE at the 10% threshold compared to the overall sample (40.3% for the second tercile, and 96.6% for the most vulnerable tercile in urban areas). For households residing in rural areas, the third tercile demonstrates a noteworthy OR of 2.077.

When comparing the most vulnerable living in urban areas across WB and GS, we see the difference between likelihood of incurring CHE at the 10% level is roughly equal. This indicates that households living in rural areas in WB may be driving the overall (full sample) results, as they are more likely to incur CHE than their urban counterparts. Additionally, population in GS is predominantly confined to densely populated urban and camp areas. Among households in refugee camps, the third tercile experiences a significant OR of 1.648.

### Index validity

Finally, as survey data contains subjective reporting of household well-being, we check whether the inclusion of self-assessed survey variables impacts the findings; results are depicted in the Supporting Information. We recreated the vulnerability index by removing any subjective factors. This definition also ensured that there are no missing households for the vulnerability index, as around 250 households did not complete the supplementary mental health survey module. The main results of our paper were largely unchanged, indicating that these subjective survey questions or missing households were not driving the findings.

## Discussion

In this paper, we have proposed a multidimensional vulnerability index to study the relationship between financial vulnerability and catastrophic healthcare expenditure (CHE) incidence in the Occupied Palestinian Territory. We have found that the most vulnerable groups are more likely to incur CHE. The consistent relationship between vulnerability and CHE implies that the index captures vulnerability according to the Palestinian “experience”, irrespective of region, thus aiding in potential policy actions.

The finding of this paper that households in the West Bank are more likely to experience CHE compared to those in the Gaza Strip is likely related to healthcare utilisation and access differences across the two regions. The impact of conflict intensity on maternal healthcare utilisation (as measured by antenatal care visits) is greater in the South West Bank compared to the Gaza strip, where antenatal care may be seen as non-necessary and stressful during times when travel is more difficult and/or dangerous [39]. Concentration of healthcare facilities is also lowest in the southern West Bank. Distance to primary (basic) healthcare services is shorter within the Gaza strip, whereas individuals in the Southern WB face checkpoints and movement restrictions more regularly. However, to access more complex and specialised procedures, Gazans usually must be moved to WB hospitals, which requires a special permit [39, 42]. Since Gazan households simply are not able to utilise these more expensive healthcare services, their risk of CHE incidence may be reduced—while at the same time, their risk of adverse health outcomes increases. For example, cardiovascular disease, the leading cause of death across the OPT in 2019, affects Gazans disproportionately compared to WB residents (49.6% of all deaths result from cardiovascular disease in Gaza compared to 29.6% in WB) (Ministry of Health, Health Annual Report, 2019). Moreover, between 2006 and 2022, Gaza’s real GDP per-capita declined by (37%), and its share in the Palestinian economy decreased from (31%) to (17.4%). Households experience a high probability of poverty (65%), labour force dropout (41%), and unemployment (45%) in this context [61].

Our puzzling finding that the possession of governmental health insurance may lead to higher likelihood of incurring CHE bears further discussion here. The current governmental health insurance scheme faces notable challenges to provision of comprehensive coverage. The scheme’s revenues only cover a small fraction (≤ 10%), of overall health service costs, highlighting a significant funding gap. Moreover, the voluntary nature of the system results in a limited subscription base, constraining its capacity to provide widespread FRP. Exemptions granted to various groups lack a compensatory mechanism, placing additional strain on the financial sustainability of the health insurance fund. The dual role of the Ministry of Health, which manages both the health insurance scheme and delivery of basic health services, creates a potential conflict of interest, impacting the quality and accessibility of health services. Costly patient transfers, often lacking defined controls and influenced by political or community pressures, further contribute to financial challenges. Additionally, long-term internal political divisions and the decision to exempt Gazans from health insurance fees have undermined expenditure control capabilities and deprive the health system of crucial revenues [62].

## Conclusions

This work has uncovered significant financial vulnerability in health among population sub-groups in the OPT. We must emphasise, however, that our data analysis represents a snapshot of the situation in 2018. We do not assess the likely increase in vulnerabilities and deterioration of financial protection that has occurred since then, due to the onset of the COVID-19 pandemic in 2020 and the continued intensification of conflict violence, culminating in the Israel-Hamas war in 2023. It is worth noting that the degree and magnitude of destruction of infrastructure, as well as deaths, injuries, and displacement from Israeli attacks on the Gaza Strip will have grave repercussions for the health system. Roughly 55% of all structures in Gaza have suffered damage, as measured by satellite imagery. As of 7 June 2024, this has amounted to: 36,731 Palestinians killed in the Gaza Strip and 83,530 Palestinians injured, including at least 12,300 children (UN-Office for the Coordination of Humanitarian Affairs). These events have probably affected the OPT population in general, but even more acutely (with future long-term repercussions) in the Gaza strip. It is plausible to expect that the collapse of the Gazan health system from late 2023 onwards will lead to a stark increase in foregone healthcare, health-related financial catastrophe, and poverty. Negative reverberations at the health system level from the overall rise in violence may be expected also in the West Bank. Future research may apply our proposed methodology to provide a rounded assessment of these consequences in due course.

We acknowledge that the cross-sectional analysis in this work could be enhanced by a panel data setting. As the SEFSec survey was run for multiple years, we leave this important and interesting extension to future work. This panel data analysis, coupled with additional information sources, would facilitate further scrutiny of the associations estimated in our analysis between CHE and potential explanatory factors (such as health insurance status), to permit firmer conclusions to be drawn about causal links. The latter would require credible inference approaches based, for instance, on the identification of valid instrumental variables, which are unavailable in the dataset used in this work. We also believe that spatial analysis using geolocated data would provide important more granular information on the implication of shocks specific to the Palestinian experience. Future work could also aim to strengthen the robustness of our vulnerability index and other indices (i.e. the multidimensional poverty index of Pinilla-Roncancio et al. (2023)) used in the analysis of determinants of CHE. We note that implementation of continuous and enhanced data monitoring mechanisms will allow for real-time identification of shifts in health vulnerabilities [63]. This proactive approach ensures that future analyses go beyond static snapshots. At this point in time, the discussion in our study still provides insights about aspects—e.g., health financing and insurance bottlenecks, sub-groups particularly vulnerable to financial risk—that will deserve careful attention in future efforts to rebuild the Palestinian health system. In particular, future policy may wish to focus on better designing insurance arrangements and strive for more informed research on FRP in humanitarian settings. These aspects will require addressing if the goal is to “build back better” the health system, avoiding the presence of left-behind sub-populations with respect to access to quality care and protection from health-related financial risks [64, 65].

## Supporting information

S1 Appendix Variable descriptions. This document describes the variables in detail and provides information on the scale of the items used in the factor analysis.
(PDF)

S1 Fig Vulnerability index number of factors. This figure presents a scree plot of the eigenvalues and a parallel analysis to confirm the correct number of factors used in creation of the vulnerability index.
(PDF)

S1 Table CHE mean differences by region. (PDF)

S2 Table Vulnerability index factor loadings. This table reports the eigenvalues, explained variance, and factor loadings from creation of the vulnerability index.
(PDF)

S3 Table Incidence of Catastrophic Health Expenditure (CHEs). (PDF)

S4 Table Catastrophic health expenditure and vulnerability (Terciles): Full coefficients. (PDF)

S5 Table Catastrophic health expenditures (Terciles), alternate specifications. (PDF)

S6 Table Catastrophic health expenditures (Quintiles), alternate specifications. (PDF)

S7 Table Catex 10%: MPCE vs vulnerability index (OR): All coefficients. (PDF)

S8 Table Catastrophic health expenditures, by insurance type: All coefficients. (PDF)

S9 Table Catastrophic health expenditures, by insurance status: All coefficients. (PDF)

S10 Table Catastrophic health expenditures, by locality type: All coefficients. (PDF)

S11 Table Catastrophic health expenditures (Quantiles) alternative composite index: Full coefficients. (PDF)

S12 Table LASSO regression of vulnerability index components: Coefficients. (PDF)

S13 Table Robustness test: Alternative regression models. (PDF)

S14 Table Robustness test: Alternative GLM specification. (PDF)
