# Supplementary material for: Multidimensional vulnerability and financial risk protection in health in contexts of protracted conflict: Evidence from the Occupied Palestinian Territory
Source: PLoS One. 2025 Jan 16;20(1):e0314852. doi: 10.1371/journal.pone.0314852 (PMC11737783; doi:10.1371/journal.pone.0314852)
Supplement: S1 Appendix — This document describes the variables in detail and provides information on the scale of the items used in the factor analysis. (PDF) [file pone.0314852.s001.pdf]

S1 Appendix: Variable Descriptions

0.1 Variable Description

- **NCDs and/or disability:** We indicate the presence of at least one HH member with Non-Communicable Diseases (NCDs) such as Diabetes mellitus, Hypertension, and Cardiovascular Diseases, and/or disability which includes difficulties in vision, hearing, movement, communication, focus, and memory. This variable is categorized into four groups:
  -(Reference group): Households in which none of the members have NCDs or disability.
  -Households with at least one member who has NCDs only.
  -Households with at least one member who has disability only.
  -Households with at least one member who has both NCDs and disabilities.
- **Employment status:** based on the household's head employment status: not working, working part-time and working full-time.
- **Household size:** A continuous variable indicating the number of HH members.
- **Insurance status:** This binary variable indicates whether the household head had insurance.
- **Insurance type:** indicates whether the household head has no health insurance at all (the reference group), has governmental health insurance only (PA only), has UNRWA health insurance only (UNRWA only), both governmental and UNRWA health insurance (PA + UNRWA) and others for other types of health insurance such as the private health insurance.
- **Educational level:** This variable is categorised into three groups indicating whether the level of education of the household head is less than secondary (the reference group), secondary or above secondary.

0.1.1 Factor Index Components

- **Self-Assessed Poverty:** Binary indicator for whether the household considers itself "poor" or "very poor"
- **Self-Assessed Financial Fragility:** Binary indicator for household either "hardly manage" or "seriously unable" to keep up financially
- **Subjective Need for Assistance:** Scale 0-2, 1 = somewhat needs assistance, 2 = high need of assistance
- **Political Conflict Shock:** Scale 0-4, equal to the total of times household answered "YES" to having witnessed or experienced the following: 1. assets/project (including land) loss/damage resulting from Israeli aggression, 2. restrictions to access to land, 3. in ability to renew work permit, or 4. exposure of any hh member to detention, injury or any other Israeli measure
- **Economic Shock:** Scale 0-6, equal to the total of times household answered "YES" to having witnessed or experienced the following: 1. assets/project (including land) loss/damage resulting from other reasons, 2. Inability to pay back a loan/debt, 3. Loss of all or total wages/salaries, 4. death of bread winner, 5. crop damage, 6. delay in getting paid a salary, 7. loss of a source of aid/assistance,
- **Health/Education Shock:** Scale 0-3, equal to the total of times household answered "YES" to having witnessed or experienced the following: 1. serious sickness, 2. in ability to attend for health treatment due to lack of medication, or 3. inability to pay for medication

- **Freedom Shock:** Scale 0-3, equal to the total of times household answered "YES" to having witnessed or experienced the following: 1. inability to leave the country for educational reasons, 2. inability to leave the country for medical treatment reasons, or 3. inability to leave the country for other reasons

- **Water Shock:** Binary indicator for having indicated experience of water shortage

- **Food Security Shock:** Scale 0-3, indicates ranked quartiles of total times and amounts having answered a series of nine questions regarding total food shortage, consumption needs, lack of food access, going to bed hungry, etc (SEFSEC questionnaire item C08).

- **Asset Ownership Index:** Scale 0-3, terciles of total amount of assets owned

- **Subjective Deprivation:** Scale 1-5, answer to the question " Do you feel deprived?" 1.Never 2. A little 3. Moderately 4. Very much 5. An extreme amount

- **Human Insecurity Scale:** Continuous 0-65, Total number according to the following scale 1.Never 2. A little 3. Moderately 4. Very much 5. An extreme amount, in answering the questions: Do you suffer physical pain? To what extent do your family fear for your personal safety? To what extent are you able to receive medical treatment when you need it? To what extent do you feel physical safety in your daily life? To what extent do you feel psychological safety in your daily life? To what extent do you fear for yourself in your daily life? To what extent do you fear for your family in your daily life? To what extent do you feel worry/ fear not being able to provide your family with daily life necessities? To what extent do you feel worry/ fear of losing your source of income or your family's source of income? To what extent do you feel worry/ fear losing your home? To what extent do you feel worry/ fear losing your land? To what extent do you feel worry/ fear about displacement and migration? To what extent do you feel worry/ fear about the chaos in the Palestinian society? To what extent do you feel worry/ fear about your future and the future of your family?
