# Supplementary material for: Multidimensional vulnerability and financial risk protection in health in contexts of protracted conflict: Evidence from the Occupied Palestinian Territory
Source: PLoS One. 2025 Jan 16;20(1):e0314852. doi: 10.1371/journal.pone.0314852 (PMC11737783; doi:10.1371/journal.pone.0314852)
Supplement: S1 Fig — This figure presents a scree plot of the eigenvalues and a parallel analysis to confirm the correct number of factors used in creation of the vulnerability index. (PDF) [file pone.0314852.s002.pdf]

PANEL A: Scree Plots of Eigenvalues

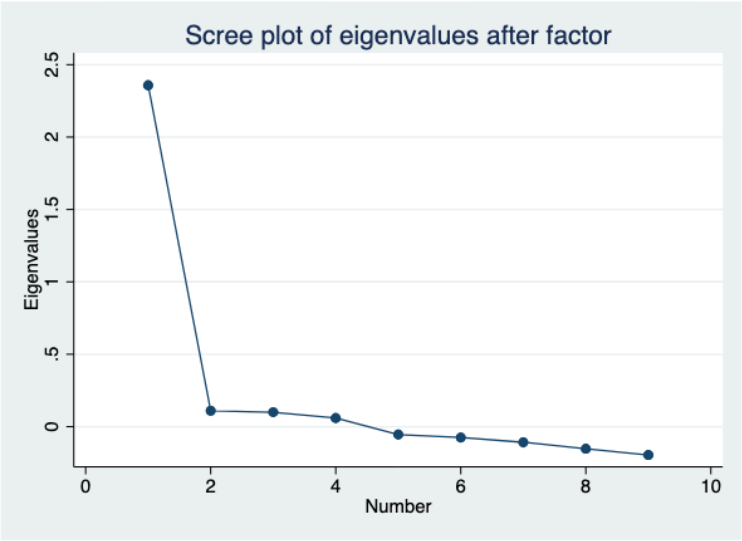

(a) WEST BANK

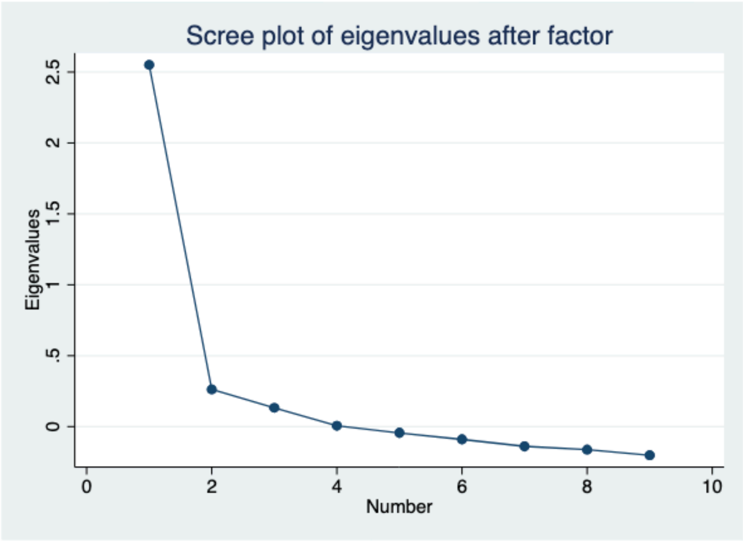

(b) GAZA STRIP

PANEL B: Parallel Analysis

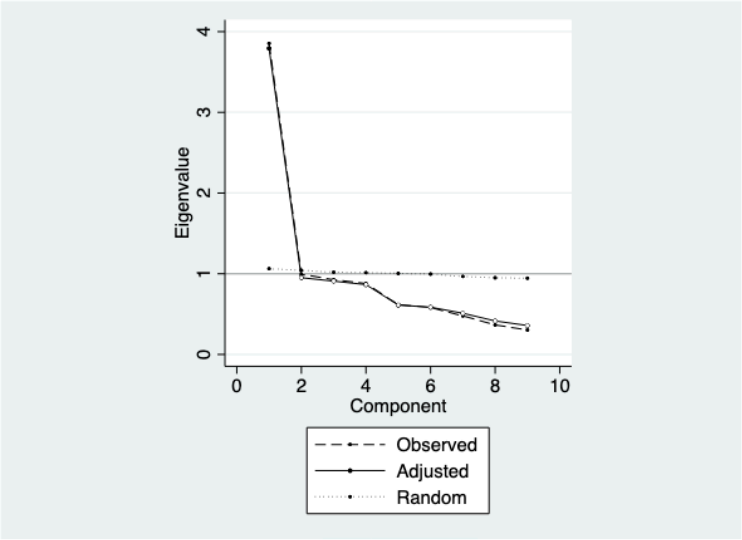

(c) WEST BANK

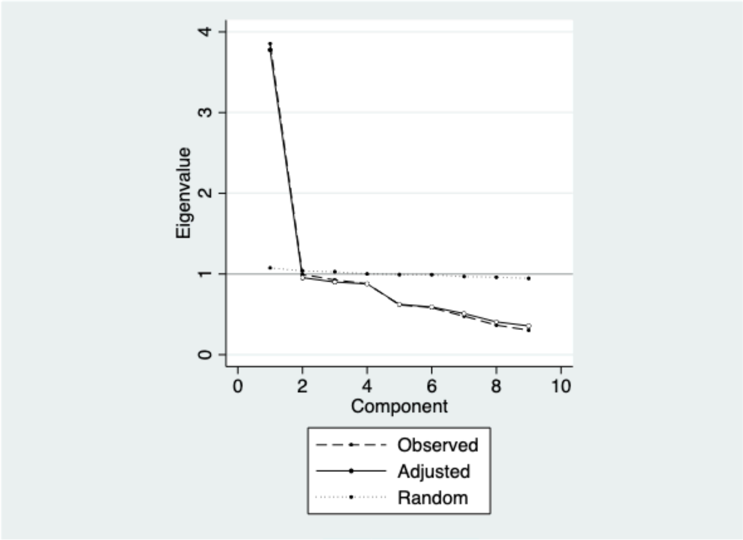

(d) GAZA STRIP

FIGURE S1. Confirming Number of Factors.
