# Supplementary material for: Multidimensional vulnerability and financial risk protection in health in contexts of protracted conflict: Evidence from the Occupied Palestinian Territory
Source: PLoS One. 2025 Jan 16;20(1):e0314852. doi: 10.1371/journal.pone.0314852 (PMC11737783; doi:10.1371/journal.pone.0314852)
Supplement: S1 Table — (PDF) [file pone.0314852.s003.pdf]

Table S1. CHE Mean differences By Region

	**CHEs Consumption Exp.**				**CHEs Nonfood Exp.**			
CHEs	WB N=5875	Gaza N=4018	Mean Diff	p-value	WB N=5874	Gaza N=4018	Mean Diff	p-value
5%	0.413	0.363	0.05***	0.000	0.583	0.502	0.08***	0.000
10%	0.191	0.171	0.02*	0.011	0.352	0.274	0.08***	0.000
15%	0.096	0.084	0.01*	0.045	0.215	0.169	0.05***	0.000
20%	0.057	0.046	0.01*	0.017	0.134	0.103	0.03***	0.000
25%	0.036	0.027	0.01*	0.016	0.090	0.064	0.03***	0.000
30%	0.024	0.017	0.01*	0.022	0.062	0.044	0.02***	0.000
35%	0.017	0.009	0.01***	0.001	0.043	0.029	0.01***	0.000
40%	0.012	0.005	0.01***	0.001	0.032	0.020	0.01***	0.000
