# Supplementary material for: Multidimensional vulnerability and financial risk protection in health in contexts of protracted conflict: Evidence from the Occupied Palestinian Territory
Source: PLoS One. 2025 Jan 16;20(1):e0314852. doi: 10.1371/journal.pone.0314852 (PMC11737783; doi:10.1371/journal.pone.0314852)
Supplement: S2 Table — This table reports the eigenvalues, explained variance, and factor loadings from creation of the vulnerability index. (PDF) [file pone.0314852.s004.pdf]

Table S2. Vulnerability Index Eigenvalues and Factor Loadings

PANEL A: Factor Analysis: West Bank (WB)

**Factor**	**Eigenvalue**	**Difference**	**Proportion**	**Cumulative**
Factor 1	2.34972	2.24654	1.1634	1.1634
Factor 2	0.10318	0.02086	0.0511	1.2145
Factor 3	0.08233	0.07043	0.0408	1.2552
Factor 4	0.01190	0.08747	0.0059	1.2611
Factor 5	-0.07557	0.03189	-0.0374	1.2237
Factor 6	-0.10747	0.04344	-0.0532	1.1705
Factor 7	-0.15090	0.04254	-0.0747	1.0958
Factor 8	-0.19344	.	-0.0958	1.0000

LR test: independent vs. saturated: χ^2 *= 9248.80, Prob* $> \chi^2$ *= 0.0000*

PANEL B: Factor Analysis: Gaza Strip (GS)

**Factor**	**Eigenvalue**	**Difference**	**Proportion**	**Cumulative**
Factor 1	2.54179	2.34759	1.1213	1.1213
Factor 2	0.19420	0.08570	0.0857	1.2069
Factor 3	0.10850	0.12263	0.0479	1.2548
Factor 4	-0.01413	0.05216	-0.0062	1.2486
Factor 5	-0.06629	0.06950	-0.0292	1.2193
Factor 6	-0.13579	0.02772	-0.0599	1.1594
Factor 7	-0.16351	0.03435	-0.0721	1.0873
Factor 8	-0.19786	.	-0.0873	1.0000

LR test: independent vs. saturated: χ^2 *= 6978.27, Prob* $> \chi^2$ *= 0.0000*

PANEL C: Factor Loadings

	WB		Gaza	
	Loadings	Uniqueness	Loadings	Uniqueness
Poverty Status (SA)	0.666	0.557	0.685	0.530
Financial Fragility (SA)	0.397	0.843	0.541	0.707
Need for Assistance (SA)	0.739	0.455	0.733	0.463
Other Shocks	0.428	0.817	0.308	0.905
Asset Ownership	0.422	0.822	0.533	0.716
Subjective Deprivation	0.535	0.714	0.544	0.704
Human Insecurity	0.184	0.966	0.233	0.946
