# Supplementary material for: Multidimensional vulnerability and financial risk protection in health in contexts of protracted conflict: Evidence from the Occupied Palestinian Territory
Source: PLoS One. 2025 Jan 16;20(1):e0314852. doi: 10.1371/journal.pone.0314852 (PMC11737783; doi:10.1371/journal.pone.0314852)
Supplement: S3 Table — (PDF) [file pone.0314852.s005.pdf]

TABLE S3. **Incidence of Catastrophic Health Expenditure (CHEs)**

	**CHEs Consumption Exp.**			**CHEs Nonfood Exp.**		
CHEs at	All	WB	Gaza	All	WB	Gaza
5%	39.28	41.31	36.31	54.99	58.27	50.20
10%	18.27	19.08	17.07	32.01	35.24	27.40
15%	9.14	9.62	8.44	19.64	21.48	16.95
20%	5.23	5.67	4.58	12.14	13.40	10.30
25%	3.20	3.56	2.69	7.96	9.01	6.42
30%	2.09	2.37	1.69	5.45	6.18	4.38
35%	1.37	1.70	0.90	3.77	4.34	2.94
40%	0.94	1.21	0.55	2.71	3.18	2.02
