# Supplementary material for: Multidimensional vulnerability and financial risk protection in health in contexts of protracted conflict: Evidence from the Occupied Palestinian Territory
Source: PLoS One. 2025 Jan 16;20(1):e0314852. doi: 10.1371/journal.pone.0314852 (PMC11737783; doi:10.1371/journal.pone.0314852)
Supplement: S4 Table — (PDF) [file pone.0314852.s006.pdf]

PANEL A: Consumption Expenditure vs. Non-food Expenditure						
Dep Var:	CHE 10%			Non-food 20%		
	(1)	(2)	(3)	(4)	(5)	(6)
Odds Ratios	All	WB	Ghaza	All	WB	Gaza
**index tercile = 2**	1.264**	1.135	1.500**	1.455***	1.420**	1.552***
	(0.141)	(0.171)	(0.251)	(0.141)	(0.234)	(0.127)
**index tercile = 3**	1.762***	1.846***	1.617***	2.053***	2.085***	1.920***
	(0.144)	(0.216)	(0.148)	(0.241)	(0.255)	(0.447)
part time	0.752**	0.690***	0.810	0.624***	0.549***	0.727
	(0.099)	(0.084)	(0.228)	(0.078)	(0.068)	(0.181)
full time	0.741***	0.699***	0.824	0.627***	0.576***	0.751***
	(0.071)	(0.092)	(0.105)	(0.055)	(0.065)	(0.083)
preparatory	0.901	0.850**	1.045	0.921	0.917	1.006
	(0.073)	(0.063)	(0.183)	(0.098)	(0.100)	(0.244)
secondary	0.774***	0.743***	0.867	0.760**	0.704**	0.913
	(0.074)	(0.065)	(0.174)	(0.083)	(0.100)	(0.168)
above secondary	0.684***	0.622***	0.793	0.711***	0.608***	0.881
	(0.074)	(0.053)	(0.179)	(0.084)	(0.054)	(0.220)
NCDs only	1.547***	1.590***	1.411***	1.761***	1.930***	1.349*
	(0.102)	(0.138)	(0.141)	(0.176)	(0.153)	(0.222)
Disability only	2.306***	2.342***	2.183***	2.615***	2.617***	2.408***
	(0.194)	(0.332)	(0.212)	(0.338)	(0.499)	(0.448)
Both	3.068***	3.355***	2.620***	3.315***	3.857***	2.521***
	(0.246)	(0.298)	(0.357)	(0.327)	(0.365)	(0.391)
PA only	1.489***	1.354***	2.528***	1.469***	1.365***	2.002***
	(0.126)	(0.089)	(0.632)	(0.111)	(0.122)	(0.364)
UNRWA only	0.975	0.962	1.450	0.930	0.968	1.041
	(0.124)	(0.147)	(0.471)	(0.121)	(0.155)	(0.308)
PA+UNRWA	1.209	1.008	2.032*	1.109	0.949	1.532
	(0.214)	(0.188)	(0.767)	(0.166)	(0.220)	(0.420)
others	0.910	0.846	1.590	0.825	0.764	1.545
	(0.282)	(0.293)	(1.438)	(0.295)	(0.314)	(1.724)
HH size	0.874***	0.872***	0.880***	0.856***	0.851***	0.869***
	(0.013)	(0.022)	(0.013)	(0.020)	(0.033)	(0.025)
Governorate FE	Yes	Yes	Yes	Yes	Yes	Yes
Observations	9647	5801	3846	9646	5800	3846
Clusters-Governorate	16	11	5	16	11	5
Log pseudolikelihood	-4155.259	-2486.087	-1654.208	-3149.32	-1939.402	-1196.529
Pseudo R^2	0.097	0.120	0.066	0.121	0.152	0.070
AIC	8340.518	4992.173	3316.416	6328.640	3898.803	2401.058
BIC	8448.134	5058.831	3341.436	6436.254	3965.459	2426.077

Exponentiated coefficients; Standard errors in parentheses. SE clustered at governorate level.
Governorate fixed effects in all models. All control variable coefficients reported in appendix.
* $p < 0.10$, ** $p < 0.05$, *** $p < 0.01$
