# Supplementary material for: Multidimensional vulnerability and financial risk protection in health in contexts of protracted conflict: Evidence from the Occupied Palestinian Territory
Source: PLoS One. 2025 Jan 16;20(1):e0314852. doi: 10.1371/journal.pone.0314852 (PMC11737783; doi:10.1371/journal.pone.0314852)
Supplement: S5 Table — (PDF) [file pone.0314852.s007.pdf]

Dep: Var: CHE-10%	(1)<br>All	(2)<br>WB	(3)<br>Gaza	(4)<br>All	(5)<br>WB	(6)<br>Gaza
Tercile =2	1.327**	1.185	1.579**	1.296**	1.157	1.549***
	(0.149)	(0.171)	(0.283)	(0.137)	(0.162)	(0.243)
Tercile =3	1.958***	2.059***	1.768***	1.891***	1.973***	1.709***
	(0.154)	(0.247)	(0.157)	(0.138)	(0.215)	(0.119)
part time	0.668***	0.650***	0.691			
	(0.091)	(0.078)	(0.205)			
full time	0.726***	0.695***	0.824			
	(0.081)	(0.098)	(0.123)			
long working hours	0.693***	0.692***	0.729*			
	(0.060)	(0.063)	(0.127)			
preparatory	0.809**	0.787**	0.891	0.794**	0.779**	0.875
	(0.073)	(0.092)	(0.133)	(0.072)	(0.090)	(0.134)
secondary	0.712***	0.674***	0.805	0.698***	0.664***	0.795
	(0.073)	(0.090)	(0.122)	(0.067)	(0.079)	(0.122)
above secondary	0.710***	0.650***	0.804*	0.686***	0.630***	0.796**
	(0.049)	(0.051)	(0.094)	(0.050)	(0.055)	(0.092)
chronic only	1.530***	1.583***	1.410***	1.547***	1.598***	1.390***
	(0.104)	(0.123)	(0.147)	(0.102)	(0.134)	(0.137)
disability only	1.779***	1.840***	1.717***	1.769***	1.808***	1.679***
	(0.196)	(0.196)	(0.335)	(0.187)	(0.175)	(0.319)
chronic and disability	2.771***	3.328***	2.128***	2.834***	3.410***	2.158***
	(0.322)	(0.464)	(0.313)	(0.300)	(0.470)	(0.236)
Official refugee status	0.806**	0.750**	0.859			
	(0.084)	(0.084)	(0.168)			
HH size	0.904***	0.902***	0.910***	0.913***	0.911***	0.921***
	(0.011)	(0.019)	(0.015)	(0.010)	(0.016)	(0.013)
one working HH member				0.712***	0.641***	0.798
				(0.063)	(0.043)	(0.123)
at least 2 working				0.631***	0.634***	0.487***
				(0.064)	(0.038)	(0.131)
PA only				1.432***	1.283***	2.568***
				(0.158)	(0.103)	(0.819)
UNRWA only				0.994	0.878	1.763*
				(0.123)	(0.118)	(0.590)
PA+UNRWA				1.133	1.008	1.997
				(0.215)	(0.206)	(0.879)
others				0.911	0.779	2.688
				(0.250)	(0.222)	(1.830)
Governorate FE	Yes	Yes	Yes	Yes	Yes	Yes
Observations	9642	5799	3843	9642	5799	3843
Clusters-Governorate	16	11	5	16	11	5
Log pseudolikelihood	-4224.132	-2521.397	-1688.526	-4213.015	-2516.139	-1675.237
Pseudo R^2	0.081	0.107	0.046	0.084	0.109	0.054
AIC	8474.263	5062.794	3385.052	8456.030	5052.279	3358.474
BIC	8567.524	5129.448	3410.068	8563.639	5118.933	3383.490

Exponentiated coefficients; Standard errors in parentheses

SE clustered at governorate level

* $p < 0.10$, ** $p < 0.05$, *** $p < 0.01$
