# Supplementary material for: Multidimensional vulnerability and financial risk protection in health in contexts of protracted conflict: Evidence from the Occupied Palestinian Territory
Source: PLoS One. 2025 Jan 16;20(1):e0314852. doi: 10.1371/journal.pone.0314852 (PMC11737783; doi:10.1371/journal.pone.0314852)
Supplement: S6 Table — (PDF) [file pone.0314852.s008.pdf]

Dep: Var: CHE-10%	(1)<br>All	(2)<br>WB	(3)<br>Gaza	(4)<br>All	(5)<br>WB	(6)<br>Gaza
Quintile=2	1.472***	1.355	1.659**	1.466***	1.358*	1.649**
	(0.218)	(0.278)	(0.405)	(0.204)	(0.252)	(0.409)
Quintile=3	1.623***	1.399	2.032***	1.596***	1.386	1.995***
	(0.213)	(0.310)	(0.269)	(0.203)	(0.305)	(0.223)
Quintile=4	1.873***	1.942***	1.730***	1.829***	1.900***	1.682***
	(0.140)	(0.215)	(0.195)	(0.129)	(0.191)	(0.187)
Quintile=5	2.580***	2.704***	2.342***	2.490***	2.602***	2.246***
	(0.239)	(0.424)	(0.151)	(0.218)	(0.378)	(0.157)
part time	0.671***	0.652***	0.695			
	(0.091)	(0.077)	(0.208)			
full time	0.730***	0.703**	0.824			
	(0.081)	(0.099)	(0.126)			
long working hours	0.698***	0.703***	0.732*			
	(0.063)	(0.066)	(0.129)			
preparatory	0.810**	0.785*	0.894	0.796**	0.779**	0.879
	(0.076)	(0.101)	(0.124)	(0.075)	(0.098)	(0.127)
secondary	0.722***	0.679***	0.823	0.709***	0.670***	0.813
	(0.074)	(0.093)	(0.117)	(0.068)	(0.083)	(0.116)
above secondary	0.726***	0.664***	0.830	0.702***	0.645***	0.821*
	(0.053)	(0.057)	(0.097)	(0.054)	(0.059)	(0.095)
chronic only	1.540***	1.594***	1.404***	1.557***	1.605***	1.385***
	(0.106)	(0.119)	(0.154)	(0.103)	(0.129)	(0.146)
disability only	1.761***	1.814***	1.707***	1.752***	1.783***	1.672***
	(0.195)	(0.201)	(0.337)	(0.185)	(0.177)	(0.320)
chronic and disability	2.760***	3.312***	2.134***	2.822***	3.386***	2.165***
	(0.318)	(0.467)	(0.309)	(0.290)	(0.458)	(0.229)
Official refugee status	0.807**	0.756**	0.855			
	(0.084)	(0.083)	(0.164)			
HH size	0.904***	0.902***	0.910***	0.913***	0.911***	0.921***
	(0.011)	(0.018)	(0.014)	(0.009)	(0.015)	(0.013)
one working member in the household				0.716***	0.647***	0.799
				(0.064)	(0.043)	(0.124)
at least 2 working members in the household				0.641***	0.646***	0.494***
				(0.066)	(0.036)	(0.134)
PA only				1.426***	1.271***	2.561***
				(0.157)	(0.101)	(0.791)
UNRWA only				0.991	0.876	1.751*
				(0.122)	(0.116)	(0.587)
PA+UNRWA				1.124	1.009	1.975
				(0.210)	(0.200)	(0.857)
others				0.910	0.782	2.819
				(0.246)	(0.218)	(1.992)
Governorate FE	Yes	Yes	Yes	Yes	Yes	Yes
Observations	9641	5798	3843	9641	5798	3843
Clusters-Governorate	16	11	5	16	11	5
Log pseudolikelihood	-4213.857	-2514.271	-1684.373	-4203.149	-2509.251	-1671.278
Pseudo R^2	0.083	0.109	0.049	0.085	0.111	0.056
AIC	8457.714	5048.541	3376.746	8436.298	5038.502	3350.556
BIC	8565.321	5115.194	3401.762	8543.905	5105.155	3375.572

Exponentiated coefficients; Standard errors in parentheses

SE clustered at governorate level

* $p < 0.10$, ** $p < 0.05$, *** $p < 0.01$
