# Supplementary material for: Multidimensional vulnerability and financial risk protection in health in contexts of protracted conflict: Evidence from the Occupied Palestinian Territory
Source: PLoS One. 2025 Jan 16;20(1):e0314852. doi: 10.1371/journal.pone.0314852 (PMC11737783; doi:10.1371/journal.pone.0314852)
Supplement: S7 Table — (PDF) [file pone.0314852.s009.pdf]

Quintile Definition:	MCPE			Vulnerability Index		
	(1)	(2)	(3)	(4)	(5)	(6)
Dep: Var: CHE-10%	All	WB	Gaza	All	WB	Gaza
**Quintle=2**	0.917	0.834*	1.088	1.448***	1.447**	1.463*
	(0.089)	(0.086)	(0.173)	(0.181)	(0.272)	(0.289)
**Quintle=3**	0.958	0.931	1.058	1.588***	1.484	1.782***
	(0.095)	(0.101)	(0.204)	(0.218)	(0.379)	(0.201)
**Quintle=4**	1.068	0.893	1.515*	1.740***	1.851***	1.571***
	(0.143)	(0.118)	(0.338)	(0.126)	(0.214)	(0.125)
**Quintle=5**	0.994	0.836	1.499	2.280***	2.486***	1.963***
	(0.160)	(0.115)	(0.462)	(0.232)	(0.398)	(0.161)
part time	0.745**	0.686***	0.813	0.756**	0.690***	0.811
	(0.101)	(0.087)	(0.226)	(0.099)	(0.083)	(0.226)
full time	0.679***	0.636***	0.775**	0.746***	0.705***	0.825
	(0.063)	(0.079)	(0.094)	(0.070)	(0.093)	(0.102)
preparatory	0.857**	0.815***	0.987	0.903	0.849**	1.046
	(0.060)	(0.060)	(0.136)	(0.074)	(0.067)	(0.175)
secondary	0.712***	0.681***	0.787*	0.788**	0.753***	0.879
	(0.060)	(0.071)	(0.101)	(0.077)	(0.070)	(0.166)
above secondary	0.587***	0.543***	0.631***	0.707***	0.643***	0.808
	(0.061)	(0.062)	(0.110)	(0.076)	(0.061)	(0.174)
NCDs only	1.508***	1.597***	1.300*	1.568***	1.616***	1.414***
	(0.105)	(0.121)	(0.188)	(0.102)	(0.135)	(0.140)
Disability only	2.504***	2.614***	2.248***	2.297***	2.341***	2.165***
	(0.237)	(0.445)	(0.224)	(0.199)	(0.338)	(0.233)
Both	3.149***	3.655***	2.455***	3.064***	3.346***	2.611***
	(0.292)	(0.387)	(0.356)	(0.251)	(0.310)	(0.357)
PA only	1.507***	1.369***	2.267***	1.491***	1.353***	2.527***
	(0.129)	(0.094)	(0.563)	(0.126)	(0.087)	(0.629)
UNRWA only	1.045	1.050	1.335	0.978	0.965	1.443
	(0.128)	(0.147)	(0.457)	(0.125)	(0.148)	(0.480)
PA+UNRWA	1.246	1.098	1.821	1.203	1.014	2.009*
	(0.207)	(0.217)	(0.665)	(0.211)	(0.186)	(0.758)
others	0.903	0.882	1.043	0.907	0.846	1.661
	(0.279)	(0.303)	(1.008)	(0.281)	(0.290)	(1.550)
HH size	0.886***	0.873***	0.924***	0.874***	0.874***	0.880***
	(0.014)	(0.021)	(0.017)	(0.012)	(0.022)	(0.012)
Governorate FE	Yes	Yes	Yes	Yes	Yes	Yes
Observations	9887	5872	4015	9647	5801	3846
Clusters-Governorate	16	11	5	16	11	5
Log pseudolikelihood	-4270.14	-2531.531	-1721.291	-4148.84	-2482.197	-1652.808
Pseudo R^2	0.091	0.115	0.062	0.098	0.122	0.067
AIC	8570.281	5083.062	3450.581	8327.681	4984.395	3313.616
BIC	8678.265	5149.841	3475.772	8435.297	5051.053	3338.635

Exponentiated coefficients; Standard errors in parentheses

SE clustered at governorate level

* $p < 0.10$, ** $p < 0.05$, *** $p < 0.01$
