# Supplementary material for: Multidimensional vulnerability and financial risk protection in health in contexts of protracted conflict: Evidence from the Occupied Palestinian Territory
Source: PLoS One. 2025 Jan 16;20(1):e0314852. doi: 10.1371/journal.pone.0314852 (PMC11737783; doi:10.1371/journal.pone.0314852)
Supplement: S8 Table — (PDF) [file pone.0314852.s010.pdf]

Insurance Type:	PA Only			UNRWA Only		
	(1)	(2)	(3)	(4)	(5)	(6)
Dep: Var: CHE-10%	All	WB	Gaza	All	WB	Gaza
**index tercile=2**	1.331**	1.133	1.876***	1.122	1.225	1.109
	(0.178)	(0.153)	(0.398)	(0.229)	(0.343)	(0.409)
**index tercile=3**	1.787***	1.877***	1.583***	1.523	1.481	1.623
	(0.172)	(0.250)	(0.219)	(0.402)	(0.519)	(0.752)
part time	0.635**	0.679	0.557***	0.379***	0.250***	0.565
	(0.113)	(0.164)	(0.120)	(0.104)	(0.079)	(0.208)
full time	0.656***	0.670**	0.657*	0.661	0.594	0.730
	(0.085)	(0.123)	(0.159)	(0.172)	(0.247)	(0.271)
long working hours	0.719***	0.713**	0.783	0.452***	0.453**	0.420***
	(0.092)	(0.098)	(0.251)	(0.067)	(0.139)	(0.021)
preparatory	0.792**	0.815	0.778	0.731	0.524	1.093
	(0.091)	(0.129)	(0.121)	(0.218)	(0.241)	(0.345)
secondary	0.757**	0.685***	0.923	0.534**	0.493*	0.604
	(0.087)	(0.073)	(0.198)	(0.130)	(0.201)	(0.198)
above secondary	0.650***	0.632***	0.726	0.723	0.635	0.906
	(0.082)	(0.090)	(0.196)	(0.143)	(0.180)	(0.203)
chronic only	1.833***	1.745***	2.091***	1.464	1.266	1.867**
	(0.166)	(0.184)	(0.445)	(0.365)	(0.491)	(0.585)
disability only	1.813***	1.753***	1.873***	1.516	1.664	1.418
	(0.258)	(0.367)	(0.430)	(0.440)	(0.790)	(0.595)
chronic and disability	3.364***	3.708***	2.520***	2.527***	2.451***	2.502***
	(0.527)	(0.769)	(0.581)	(0.514)	(0.723)	(0.586)
HH size	0.932***	0.930**	0.939**	0.880***	0.902	0.865***
	(0.020)	(0.031)	(0.028)	(0.036)	(0.076)	(0.021)
Governorate FE	Yes	Yes	Yes	Yes	Yes	Yes
Observations	3246	2212	1034	1352	728	624
Clusters-Governorate	16	11	5	16	11	5
Log pseudolikelihood	-1550.178	-1046.168	-496.7447	-513.2543	-269.3285	-240.6537
Pseudo R^2	0.100	0.119	0.070	0.108	0.152	0.065
AIC	3124.355	2112.335	1001.489	1050.509	558.657	489.307
BIC	3197.377	2169.352	1021.254	1113.021	604.560	507.052

Exponentiated coefficients; Standard errors in parentheses

SE clustered at governorate level

* $p < 0.10$, ** $p < 0.05$, *** $p < 0.01$
