# Supplementary material for: Multidimensional vulnerability and financial risk protection in health in contexts of protracted conflict: Evidence from the Occupied Palestinian Territory
Source: PLoS One. 2025 Jan 16;20(1):e0314852. doi: 10.1371/journal.pone.0314852 (PMC11737783; doi:10.1371/journal.pone.0314852)
Supplement: S9 Table — (PDF) [file pone.0314852.s011.pdf]

Insurance Status:		**Insured**			**Uninsured**	
	(1)	(2)	(3)	(4)	(5)	(6)
Dep: Var: CHE-10%	All	WB	Gaza	All	WB	Gaza
**index tercile=2**	1.334**	1.121	1.620***	1.337*	1.337	1.312
	(0.172)	(0.168)	(0.300)	(0.226)	(0.236)	(1.010)
**index tercile=3**	1.825***	1.820***	1.767***	2.622***	2.571***	2.339
	(0.172)	(0.307)	(0.157)	(0.356)	(0.343)	(2.540)
part time	0.638***	0.633***	0.644	0.815	0.693*	2.907
	(0.099)	(0.089)	(0.195)	(0.167)	(0.132)	(2.418)
full time	0.730***	0.722*	0.773	0.798	0.690***	3.116**
	(0.089)	(0.134)	(0.124)	(0.114)	(0.092)	(1.784)
long working hours	0.674***	0.665***	0.717**	0.850	0.770	1.322
	(0.072)	(0.104)	(0.116)	(0.141)	(0.131)	(1.298)
preparatory	0.835*	0.837	0.875	0.664***	0.653***	1.050
	(0.086)	(0.128)	(0.128)	(0.084)	(0.088)	(0.595)
secondary	0.704***	0.672***	0.771*	0.641**	0.623**	0.700
	(0.072)	(0.087)	(0.116)	(0.112)	(0.116)	(0.503)
above secondary	0.713***	0.635***	0.814	0.533***	0.566**	0.261*
	(0.063)	(0.076)	(0.105)	(0.119)	(0.139)	(0.212)
chronic only	1.414***	1.496***	1.317***	1.934***	1.770***	5.743**
	(0.110)	(0.155)	(0.120)	(0.309)	(0.274)	(4.486)
disability only	1.689***	1.821***	1.587**	1.890**	1.631*	5.204**
	(0.200)	(0.217)	(0.301)	(0.547)	(0.476)	(3.871)
chronic and disability	2.582***	3.204***	1.996***	3.221***	3.459***	2.034
	(0.304)	(0.459)	(0.303)	(0.778)	(0.778)	(3.718)
HH size	0.902***	0.903***	0.906***	0.897***	0.894***	0.909
	(0.012)	(0.021)	(0.016)	(0.024)	(0.025)	(0.110)
Governorate FE	Yes	Yes	Yes	Yes	Yes	Yes
Observations	7670	4063	3607	1972	1736	236
Clusters-Governorate	16	11	5	16	11	5
Log pseudolikelihood	-3431.259	-1808.249	-1611.824	-769.8006	-706.5768	-56.89859
Pseudo R^2	0.079	0.110	0.048	0.111	0.105	0.197
AIC	6886.518	3636.498	3231.649	1565.601	1435.154	121.797
BIC	6969.859	3699.595	3256.411	1638.230	1495.206	135.653

Exponentiated coefficients; Standard errors in parentheses

SE clustered at governorate level

* $p < 0.10$, ** $p < 0.05$, *** $p < 0.01$
