# Supplementary material for: Multidimensional vulnerability and financial risk protection in health in contexts of protracted conflict: Evidence from the Occupied Palestinian Territory
Source: PLoS One. 2025 Jan 16;20(1):e0314852. doi: 10.1371/journal.pone.0314852 (PMC11737783; doi:10.1371/journal.pone.0314852)
Supplement: S10 Table — (PDF) [file pone.0314852.s012.pdf]

Insurance Status:	Urban			Rural	Camps
	(1)	(2)	(3)	(4)	(5)
Dep: Var: CHE-10%	All	WB	Gaza	All	All
Index Tercile =2	1.403***	1.212	1.740***	0.987	1.115
	(0.157)	(0.161)	(0.271)	(0.245)	(0.179)
Index Tercile =3	1.966***	1.979***	1.987***	2.077**	1.648**
	(0.112)	(0.184)	(0.136)	(0.591)	(0.384)
part time	0.705**	0.664***	0.737	0.718*	0.391***
	(0.113)	(0.080)	(0.248)	(0.133)	(0.131)
full time	0.735***	0.728**	0.756**	0.586**	0.965
	(0.083)	(0.118)	(0.107)	(0.131)	(0.227)
long working hours	0.695***	0.709***	0.688*	0.609*	1.125
	(0.065)	(0.067)	(0.137)	(0.165)	(0.248)
preparatory	0.867	0.818	0.973	0.729***	0.481***
	(0.097)	(0.124)	(0.166)	(0.064)	(0.076)
secondary	0.738***	0.685**	0.841	0.599***	0.646**
	(0.084)	(0.109)	(0.131)	(0.086)	(0.114)
above secondary	0.723***	0.629***	0.876	0.671	0.511**
	(0.063)	(0.090)	(0.082)	(0.174)	(0.150)
chronic only	1.535***	1.537***	1.525***	1.294	1.236
	(0.157)	(0.215)	(0.229)	(0.291)	(0.470)
disability only	1.624***	1.659***	1.564*	2.234***	1.971***
	(0.247)	(0.264)	(0.378)	(0.461)	(0.456)
chronic and disability	2.762***	3.402***	2.197***	2.389***	1.807*
	(0.328)	(0.535)	(0.286)	(0.662)	(0.621)
HH size	0.904***	0.895***	0.914***	0.916***	0.821***
	(0.014)	(0.026)	(0.020)	(0.023)	(0.048)
PA only	1.408**	1.243	2.576***	1.449***	1.085
	(0.213)	(0.168)	(0.856)	(0.187)	(0.586)
UNRWA only	0.907	0.819	1.593	1.207	1.276
	(0.153)	(0.187)	(0.622)	(0.214)	(0.599)
PA+UNRWA	1.025	0.888	1.824	1.126	1.217
	(0.215)	(0.220)	(0.830)	(0.191)	(0.552)
others	0.828	0.679	2.839	1.492	0.201
	(0.288)	(0.232)	(2.241)	(0.526)	(0.257)
Governorate FE	Yes	Yes	Yes	Yes	Yes
Observations	7253	4008	3245	1417	972
Clusters-Governorate	16	11	5	11	14
Log pseudolikelihood	-3179.821	-1767.409	-1398.255	-585.0798	-382.3032
Pseudo R^2	0.084	0.110	0.056	0.143	0.137
AIC	6389.642	3554.817	2804.510	1190.160	790.606
BIC	6492.979	3617.778	2828.850	1242.723	854.038

Exponentiated coefficients; Standard errors in parentheses

SE clustered at governorate level

* $p < 0.10$, ** $p < 0.05$, *** $p < 0.01$
