# Supplementary material for: Multidimensional vulnerability and financial risk protection in health in contexts of protracted conflict: Evidence from the Occupied Palestinian Territory
Source: PLoS One. 2025 Jan 16;20(1):e0314852. doi: 10.1371/journal.pone.0314852 (PMC11737783; doi:10.1371/journal.pone.0314852)
Supplement: S11 Table — (PDF) [file pone.0314852.s013.pdf]

Dep: Var: CHE-10%<br>Odd-ratios	(1)<br>All	(2)<br>WB	(3)<br>Gaza
Index Quintile New=2	1.447***	1.337	1.634**
	(0.207)	(0.244)	(0.409)
Index Quintile New=3	1.582***	1.374	1.989***
	(0.202)	(0.291)	(0.260)
Index Quintile New=4	1.792***	1.877***	1.678***
	(0.136)	(0.186)	(0.246)
Index Quintile New=5	2.431***	2.540***	2.286***
	(0.213)	(0.343)	(0.203)
part time	0.683***	0.669***	0.693
	(0.094)	(0.073)	(0.216)
full time	0.749***	0.727**	0.817
	(0.084)	(0.100)	(0.136)
long working hours	0.728***	0.736***	0.742
	(0.068)	(0.067)	(0.140)
preparatory	0.820**	0.798*	0.897
	(0.080)	(0.107)	(0.129)
secondary	0.727***	0.684***	0.827
	(0.075)	(0.088)	(0.130)
above secondary	0.721***	0.660***	0.840
	(0.054)	(0.061)	(0.100)
chronic only	1.488***	1.546***	1.347***
	(0.098)	(0.110)	(0.152)
disability only	1.699***	1.753***	1.633**
	(0.192)	(0.195)	(0.336)
chronic and disability	2.606***	3.150***	2.024***
	(0.288)	(0.449)	(0.261)
PA only	1.397***	1.240**	2.583***
	(0.156)	(0.108)	(0.793)
UNRWA only	0.987	0.970	1.682
	(0.119)	(0.160)	(0.552)
PA+UNRWA	1.098	1.071	1.868
	(0.195)	(0.241)	(0.786)
others	0.921	0.810	2.803
	(0.246)	(0.221)	(1.943)
Rural	1.119	1.076	
	(0.162)	(0.159)	
Camps	0.971	0.612	1.238
	(0.193)	(0.193)	(0.261)
HH size	0.900***	0.897***	0.906***
	(0.011)	(0.019)	(0.013)
received any assistance	1.150**	1.216*	1.108
	(0.076)	(0.135)	(0.104)
Governorate FE	Yes	Yes	Yes
Observations	9641	5798	3843
Clusters-Governorate	16	11	50
Log pseudolikelihood	-4202.069	-2504.972	-1672.698
Pseudo R^2	0.086	0.113	0.055
AIC	8434.138	5029.943	3353.396
BIC	8541.744	5096.596	3378.412

Exponentiated coefficients; Standard errors in parentheses

SE clustered at governorate level

* $p < 0.10$, ** $p < 0.05$, *** $p < 0.01$
