# Supplementary material for: Multidimensional vulnerability and financial risk protection in health in contexts of protracted conflict: Evidence from the Occupied Palestinian Territory
Source: PLoS One. 2025 Jan 16;20(1):e0314852. doi: 10.1371/journal.pone.0314852 (PMC11737783; doi:10.1371/journal.pone.0314852)
Supplement: S12 Table — (PDF) [file pone.0314852.s014.pdf]

	(1) WB	(2) Gaza	(3) WB	(4) Gaza
Poverty Status (SA)	0.0693	**X**	**X**	-0.0692
Financial Fragility (SA)	0.212	0.00467	0.158	**X**
Need for Assistance (SA)	0.216	0.0416	0.172	0.00510
Other Shocks	0.314	0.279	-	-
Asset Ownership	0.431	0.217	0.420	0.151
Subjective Deprivation	-0.0501	0.0890	-0.0426	0.0522
Human Insecurity	-0.00782	-0.0179	-0.00523	-0.0179
Environmental Shock			-0.142	-0.135
Health and Education Shock			0.576	0.577
Freedom Shock			-0.0371	0.246
Water Shock			0.439	0.0563
Political Conflict Shock			**X**	-0.0695
Constant	-2.862	-2.204	-2.568	-1.692
N	5820	3848	5820	3848

Coefficients from a LASSO regression of catastrophic health expenditure at 10% of OOP.

X indicates the lasso did not select the variable for inclusion.

Columns (3) and (4) use the individual components of “Other Shocks” in the regression.
