# Supplementary material for: Multidimensional vulnerability and financial risk protection in health in contexts of protracted conflict: Evidence from the Occupied Palestinian Territory
Source: PLoS One. 2025 Jan 16;20(1):e0314852. doi: 10.1371/journal.pone.0314852 (PMC11737783; doi:10.1371/journal.pone.0314852)
Supplement: S13 Table — (PDF) [file pone.0314852.s015.pdf]

MODEL:	(1) (Fixed)	(2) (Mixed)	(3) (GLM)	(4) (xtlogit)
Index Tercile =2	1.292**	1.302***	1.381***	1.292***
	(0.139)	(0.097)	(0.157)	(0.096)
Index Tercile =3	1.850***	1.870***	1.973***	1.850***
	(0.141)	(0.141)	(0.136)	(0.140)
part time	0.681***	0.685***	0.708**	0.681***
	(0.093)	(0.068)	(0.100)	(0.068)
full time	0.747***	0.750***	0.774**	0.747***
	(0.084)	(0.059)	(0.083)	(0.059)
long working hours	0.724***	0.725***	0.738***	0.724***
	(0.065)	(0.060)	(0.068)	(0.060)
preparatory	0.819**	0.816***	0.797**	0.819***
	(0.078)	(0.058)	(0.072)	(0.058)
secondary	0.718***	0.714***	0.694***	0.718***
	(0.073)	(0.062)	(0.069)	(0.063)
above secondary	0.707***	0.702***	0.676***	0.707***
	(0.051)	(0.059)	(0.050)	(0.060)
chronic only	1.476***	1.474***	1.447***	1.476***
	(0.096)	(0.121)	(0.094)	(0.122)
disability only	1.713***	1.701***	1.621***	1.713***
	(0.192)	(0.156)	(0.181)	(0.157)
chronic and disability	2.607***	2.600***	2.545***	2.607***
	(0.291)	(0.236)	(0.286)	(0.237)
PA only	1.401***	1.386***	1.260	1.401***
	(0.158)	(0.113)	(0.193)	(0.114)
UNRWA only	0.988	0.961	0.798	0.988
	(0.122)	(0.104)	(0.155)	(0.107)
PA+UNRWA	1.103	1.070	0.887	1.103
	(0.200)	(0.106)	(0.231)	(0.110)
others	0.920	0.940	0.891	0.920
	(0.249)	(0.155)	(0.252)	(0.156)
Rural	1.118	1.103	0.973	1.118
	(0.165)	(0.094)	(0.159)	(0.097)
Camps	0.971	0.977	0.948	0.971
	(0.192)	(0.098)	(0.190)	(0.098)
HH size	0.899***	0.900***	0.905***	0.899***
	(0.011)	(0.010)	(0.011)	(0.010)
received assistance	1.177**	1.155*	1.071	1.177**
	(0.081)	(0.090)	(0.095)	(0.095)
Governorate FE	Yes	No	No	Yes
Observations	9642	9642	9642	9642
Log pseudolikelihood	-4211.693		-4276.831	
Log likelihood		-4234.216		-4211.693
Pseudo R^2	0.084			
AIC	8453.387	8510.432	8583.662	8495.387
BIC	8560.995	8661.083	8691.271	8753.647

Exponentiated coefficients; Standard errors in parentheses

SE clustered at governorate level

* $p < 0.10$, ** $p < 0.05$, *** $p < 0.01$
