# Supplementary material for: Multidimensional vulnerability and financial risk protection in health in contexts of protracted conflict: Evidence from the Occupied Palestinian Territory
Source: PLoS One. 2025 Jan 16;20(1):e0314852. doi: 10.1371/journal.pone.0314852 (PMC11737783; doi:10.1371/journal.pone.0314852)
Supplement: S14 Table — (PDF) [file pone.0314852.s016.pdf]

Dep. Var: CHE-10%	glamm
Index Tercile== 1	0.545***
	(0.041)
Index Tercile== 2	0.706***
	(0.047)
Not working	1.395***
	(0.095)
Working part time	0.924
	(0.090)
elementary or less	1.433***
	(0.120)
preparatory education	1.148*
	(0.096)
secondary education	1.010
	(0.098)
no disability or chronic	0.476***
	(0.034)
chronic only	0.696***
	(0.063)
no insurance	0.987
	(0.086)
PA only	1.375***
	(0.090)
Urban	0.950
	(0.064)
HH size	0.898***
	(0.010)
Received any type of assistance	1.167**
	(0.090)
Governorate FE	No
Observations	9642
Log likelihood	-4243.387
AIC	8518.774
BIC	8633.556

Exponentiated coefficients; Standard errors in parentheses

SE clustered at governorate level

* $p < 0.10$, ** $p < 0.05$, *** $p < 0.01$
